# Bcl-2-associated athanogene 3(BAG3) is associated with tumor cell proliferation, migration, invasion and chemoresistance in colorectal cancer

**DOI:** 10.1186/s12885-018-4657-2

**Published:** 2018-08-06

**Authors:** Ning Li, Minghong Chen, Yansha Cao, Hua Li, Jinping Zhao, Zhenhua Zhai, Fu Ren, Keyan Li

**Affiliations:** 1 Department of Biochemistry and Molecular Biology,College of Basic Medicine, Jinzhou Medical University, Jinzhou, 121000 Liaoning China; 2 The Laboratory of Tumor Angiogenesis and Microenvironment, The First Hospital Affiliated to Jinzhou Medical University, Jinzhou, 121000 Liaoning China; 3 Department of Oncology, Cancer Centre, The First Affiliated Hospital of Jinzhou Medical University, Jinzhou, 121000 Liaoning China; 4 Department of Biological Anthropology Institute, College of Basic Medicine, Jinzhou Medical University, Jinzhou, 121000 Liaoning China; 5 Department of Cardiology, the First Affiliated Hospital of Jinzhou Medical University, Jinzhou Medical University, No. 2, Section 5, Renmin Road, Ling he District, Jinzhou, Liaoning 121000 People’s Republic of China

**Keywords:** Colorectal cancer, HCT-116, BAG3, Proliferation, Migration, Invasion, Chemoresistance, Microarray, Gene-interaction network

## Abstract

**Background:**

CRC is one of the most common malignancies worldwide, and its molecular mechanisms remain unclear. Elevated levels of BAG3 have been reported in various tumors. The present study aimed to explore the expression and function of BAG3 in CRC.

**Methods:**

BAG3 protein expression was evaluated in 90 CRC specimens using immunohistochemistry in tissue microarrays, and the correlation between BAG3 expression and the clinicopathological features were assessed. In HCT116 cells BAG3 overexpression cell models were constructed, and CRISPR/Cas9 was used for BAG3 knockout. Western blotting and quantitative real-time PCR were used to determine BAG3 expression in HCT-116 Cells. Cell proliferation, migration and invasion were analyzed by cell counting, colony formation assay, EdU cell proliferation assay, RTCA growth curve assays, wound-healing migration assay and transwell invasion assay. The influence of BAG3 expression level on chemoresistance in HCT-116 cells was examined. Gene expression microarray and IPA analyses were employed to explore signaling pathways associated with the control of BAG3.

**Results:**

Using immunohistochemistry, this study found that BAG3 was markedly upregulated in colorectal cancer tissues and that BAG3 levels were significantly associated with tumor size and gender. BAG3 overexpression promoted HCT-116 cell growth, migration and invasion in vitro. In contrast, BAG3 knockout inhibited HCT-116 cell growth, migration and invasion. HCT-116 cells with high expression of BAG3 had higher cell viability and lower apoptosis rate than control cells after treatment with 5-FU, while the BAG3 knockout group demonstrated the opposite effects. So BAG3 expression level was associated with chemoresistance to 5-FU in HCT-116 cells. Gene expression microarrays and bioinformatics analyses of HCT-116 cells with BAG3 knockout demonstrated the involvement of BAG3 in signaling pathways associated with the control of cell proliferation, migration, invasion and chemoresistance in CRC.

**Conclusions:**

In conclusion, this study provided evidence that BAG3 has a relevant role in CRC biology, and defined potential molecular pathways and networks. So BAG3 may be considered as a potential therapeutic target for anti-tumor therapy in colorectal cancer.

## Background

Colorectal cancer (CRC), one of the most common malignancies worldwide, is the second most common cause of cancer-related deaths in developed countries [1, 2]. With incidence steadily rising in recent years, CRC has become the third most frequent cancer in men and the second most frequent cancer in women [3]. Despite advances in cancer diagnosis and treatment, the survival rate for patients who suffer from CRC remains poor [3]. Therefore, a more detailed understanding of CRC molecular mechanisms is necessary to develop more effective therapies.

CRC carcinogenesis develops in a multi-step process, which is based on excessive cell proliferation and the inhibition of apoptosis [4]. Bcl-2 associated athanogene 3 (BAG3) is an anti-apoptosis protein that was highly conserved throughout evolution and is primarily distributed in the cytoplasm. The BAG3 expression level is usually low or barely detectable in most normal tissues (except for the cardiac and skeletal muscle tissues); however, high BAG3 expression levels are detected in many solid tumors, such as prostate cancer, ovarian cancer, and glioblastoma [5–8]. Stress conditions, such as high temperatures, HIV infection, the presence of heavy metals (Zn or Cd), and proteasome inhibitors can increase BAG3 expression [9–12]. As a co-chaperone, BAG3 interacts with the molecular chaperone heat shock protein 70 (HSP70) to participate in a wide range of cellular processes, including apoptosis, cell remodeling, and autophagy [13, 14]. BAG3 protein induces endothelial vascular remodeling and tumor formation via the phosphorylation of ERK (extracellular signal-related kinase) in the Raf/MEK/ERK pathway [15]. The BAG3 protein is also involved in cell proliferation, adhesion, migration, invasion, and epithelial mesenchymal transition (EMT) [16, 17]. Studies have also indicated that BAG3 contributes to autophagy regulation [18–23].

However, the role of BAG3 in the progression of CRC remains unclear. Therefore, this study examined the relationship between BAG3 and CRC. The study found that BAG3 expression was upregulated in CRC tissues and associated with clinicopathological features. BAG3 overexpression in HCT-116 cells promoted cell proliferation, migration, and invasion in vitro, while BAG3 knockout inhibited these processes. BAG3 also caused chemoresistance to 5-fluorouracil in HCT-116 cells. Gene expression microarrays and bioinformatics analysis of HCT-116 cells with BAG3 knockout demonstrated the involvement of BAG3 in signaling pathways associated with the control of cell biology in CRC. The cumulative BAG3 observations noted indicate the major BAG3-mediated signaling networks involved in CRC and point to BAG3’s possible role in tumorigenesis.

## Methods

### Cell lines and culture

The human colorectal cancer cell line HCT-116 cell was purchased from Nanjing KeyGenBiotech Inc. (Nanjing, China) and grown in HyClone-Dulbecco’s modified eagle medium (DMEM) containing 10% fetal bovine serum (HyClone, Beijing, China) at 37 °C in a humidified incubator with 5% CO_2_.

### Lentiviruses infection for BAG3 stable cell lines

Human Lenti-BAG3-EGFPs and Lenti-vector controls were designed and purchased from the GeneChem Corporation (Shanghai, China), and transfections were performed according to the standard procedure. HCT-116 cells were seeded in six-well plates (2 × 10^5^ cells/well) containing DMEM supplement with 10% fetal bovine serum (FBS). Cells were then infected with Lenti-BAG3-EGFP and Lenti-vector control. Following lentiviruses infection, single cell clones were selected in the presence of puromycin for 2 to 4 weeks.

### CRISPR/Cas9 knockout of BAG3 gene

The CRISPR/Cas9 double vector lentiviruses for BAG3 gene knockout were designed and synthesized by GeneChem Corporation (Shanghai, China). HCT-116 cells were transduced with lentiviruses expressing Cas9 nuclease, and stably transduced cells were selected with 1 μg/ml puromycin. BAG3 lentiviruses were then transfected into HCT-116-Cas9 stable cell line for BAG3 knockout. After 72 h, DNA was extracted from the transfected cells using a DNA extraction kit (QIAGEN, Germany) according to the manufacturer’s instructions, and proteins were extracted with RIPA buffer. BAG3 expression was examined by fluorescence microscope, agarose gel electrophoresis and Western blot.

### RNA isolation and quantitative real-time PCR

The extraction of RNA and quantitative real-time PCR (qRT-PCR) was performed as described previously [24]. For BAG3 qRT-PCR analysis, the following primers were used: forward, 5’-ATGCGCGATTCCGAACTGAG-3′ and reverse, 5’-AGGATGAGCAGTCAGAGGCAG-3′; additionally, 18S rRNA was used as an endogenous control: forward, 5’-AATAGCCTTTGCCATCAC-3′ and reverse, 5’-CGTTCCACCTCATCCTC-3′. BAG3 mRNA expression was analyzed using a real-time PCR instrument, TP800 (Takara, Japan). The relative BAG3 expression was normalized to 18S rRNA, and data analyses were performed using the comparative CT method [25].

### Colony formation assay

For the colony formation assay, HCT-116 cells with BAG3 overexpression or knockout were cultured to reach the logarithmic growth phase. Cells were seeded into three 10 cm diameter plates at a density of 50/100/200 cells per plate and cultured for 2 weeks. Cell colonies were stained with crystal violet, counted under a microscope and quantified using ImageJ (National Institutes of Health, Bethesda, USA). Colonies with a diameter more than 100 μm were counted.

### EdU cell proliferation assay

5-Ethynyl-20-deoxyuridine (EdU) cell proliferation assay was conducted to detect cell proliferation. According to EdU kit instructions (Life Technologies, USA), HCT-116 cells were inoculated into a 24-well plate at a density of 1 × 10^5^ cells per well and cultured for 6 h, incubated with 100 μl of 50 μM EdU solution for 2 h, fixed in 4% formaldehyde, observed under an Olympus fluorescence microscope (Tokyo, Japan) and photographed.

### Growth curve assay using real-time cell analyzer (RTCA)

Growth curve assay was performed by using real-time cell analyzer (RTCA) with the xCELLigence system (ACEA Bioscience, San Diego, CA) according to the manufacturer’s instructions. To monitor cells continuously, the cells were seeded in the RTCA E-plates (ACEA Bioscience, San Diego, CA) at a density of 1 × 10^4^ cells per well, after incubated for 1 h, the baseline was detected, and then the electrical impedance in each well was measured continuously for nearly 3 days. The shift of the electrical impedance is expressed as the cell index, which is a parameter of cell viability.

### Migration and invasion assays

For wound-healing migration assay, cells were seeded in 6-well plates for 24 h, and then 200 μl pipette tip was used to scratch the cells. The cells were then washed with phosphate-buffered saline (PBS) and cultured in Dulbecco’s modified eagle medium with FBS-free medium for 24 and 48 h. Wounds were observed under a microscope and photographed at 0/24/48 h.

The transwell invasion assay was performed using BD BioCoat Matrigel invasion chambers (8-μm pore size, BD, USA). The filters were pre-coated with 100 μl Matrigel at 1:4 dilution in DMEM to form a genuine reconstituted basement, then 600 μl DMEM medium containing 10% FBS was added to the lower chamber, and 1 × 10^4^ cells /100 μl serum-free media were placed into the upper chamber of the transwell insert. After incubation for 24 h, cells remaining on the upper membrane were removed with a cotton swab, while cells that had invaded through the membrane were fixed in formaldehyde, stained with crystal violet and counted using an Olympus fluorescence microscope (Tokyo, Japan).

### Immunohistochemical analysis

Ninety colorectal cancer tissue microarrays (HCol-Ade180Sur-04) were purchased from Shanghai Outdo Biotech Co., Ltd. (Shanghai, China). The clinicopathological characteristics of the samples were available on the company’s website. The patients were all pathologically diagnosed with colon cancer and consented with this study. These patients were operated between July 2006 and May 2007, and the last follow-up was August 2013. Immunohistochemical analysis was performed as previously described [26]. The tissue array slides were deparaffinized in xylene, rehydrated in 100, 95, and 75% ethanol, and antigen was retrieved with a citrate buffer. A 3% hydrogen peroxide solution was then used to quench the endogenous peroxidase. Goat serum was then used to block non-specific binding. The slides were probed with BAG3 primary antibody (1:100, Cat # NBPI-866442, Novus Biologicals, USA) at 4 °C overnight and then incubated with secondary antibody (1:5000, rabbit, Amersham Biosciences) followed by DAB kit (Life Technologies, USA).The PBS solution was used as negative control.

The intensity and percentage of immunoreactive cells (immunoreactive score, IRS system) were used to score the immunohistochemical staining. Immunostaining intensity was scored as 0–3 (0, negative; 1, weak; 2, moderate; 3, strong), while the percentage of immunoreactive cells was scored as 1–4 (1, 0–25%; 2, 26–50%; 3, 51–75%; 4, 76–100%). The multiplication of these two scores for each sample was used to generate the final IRS score. Finally, the immunoreactivity of the stained tissue samples was classified into low (0–5 scores) or high expression (6–12 scores), respectively.

### Western blot analysis

Western blot analysis was performed as previously described [26]. Total protein levels were measured using BCA protein assay kit (Pierce, USA). 20 μg of total protein was mixed with 2× loading buffer and separated by 10% SDS-PAGE. Proteins were transferred onto a Hybond polyvinylidene difluoride membrane (Millipore, USA). After overnight incubation at 4 °C with the BAG3 primary antibody (1:1000, Cat # MABC276, Clone AC-2, Millipore, USA) and GAPDH antibody (1:2000, rabbit, Amersham Biosciences), membranes were washed with TBST and incubated with the secondary antibody (1:5000, mouse, rabbit, Amersham Biosciences) at room temperature for 1 h. Finally, bands were captured using Image Quant LAS4000 (GE, USA).

### Chemoresistance assay

To evaluate the differences in chemoresistance properties according to the expression levels of BAG3 in HCT-116 cells, cells were seeded in 96-well plates with 80–90% confluence and treated with 0,5,25,50 μg/ml 5-FU, the concentrations used were determined by referring to previous report [27]. 5-FU was dissolved in DMSO (stock concentration 50 mg/ml) and a starting working concentration used in experiments was 5 mg/ml which was further diluted 1000-fold, 200-fold and 100-fold to 5 μg /ml, 25 μg /ml and 50 μg /ml. After 24 h or 48 h, relative cell viability was detected by MTT assay, and apoptotic cells were determined by flow cytometry. For the MTT assay, 50 μl of MTT solution was added to each well followed by incubation at room temperature for 2 h. The cells were then updated with the addition of 150 μl DMSO to dissolve the precipitate. Optical density was measured at 570 nm using a spectrophotometer (Bio-Rad, USA). Flow cytometric analysis of apoptosis was performed using an Annexin V-PE and 7-AAD apoptosis detection kit (Tianjin Sungene Biotech Co., Ltd., China).The cells were harvested, washed with PBS and suspended in binding buffer. Then, 5 μl annexinV-PE was added to 100 μl of the cell suspension, which was incubated at room temperature for 15 min, protected from light. Finally 5 μl 7-AAD was added 5 min prior to detection using a flow cytometer (FACSCalibur, BD, USA).

### Gene expression microarray analysis

Total RNA was extracted from HCT-116 cells infected with lentiviruses expressing either Scr-shRNA (*n* = 3) or BAG3-shRNA (*n* = 3) using Trizol reagent (Thremo, USA). NanoDrop 2000 (Thremo, USA) and an Agilent Bioanalyzer 2100 (Agilent, USA) were used to detect RNA quantity and quality, respectively. Gene expression analysis was determined using the Affymetrix GeneChip PrimeView Human Gene Expression Array (Affymetrix, USA). Raw data were collected using the GeneChip Scanner 3000 (Affymetrix, USA) for array scanning. Genes with significantly altered expression were selected based on the following criteria: *P* < 0.05 and absolute fold change > 2. Pathway enrichment analysis was performed for all significant differential genes based on Ingenuity Pathway Analysis (IPA) (QIAGEN, Germany).

### Statistical analysis

The data analyses were conducted using SPSS 17.0 (SPSS Inc., Chicago, USA). Data are presented as the mean ± SD, and statistical analyses were performed using the Chi-square test and ANOVA where appropriate. The different expression of BAG3 between cancer and adjacent non-tumor tissues was analyzed by χ^2^ test. The association between BAG3 expression and clinicopathological features was explored suing Spearman rank correlation. Kaplan-Meier method was used to estimate survival rates. A multivariate analysis of independent prognostic factors was conducted using the Cox proportional hazards model. *P* < 0.05 indicated statistical significance.

## Results

### BAG3 protein is overexpressed in human colorectal cancer tissues

**Fig. 1:**
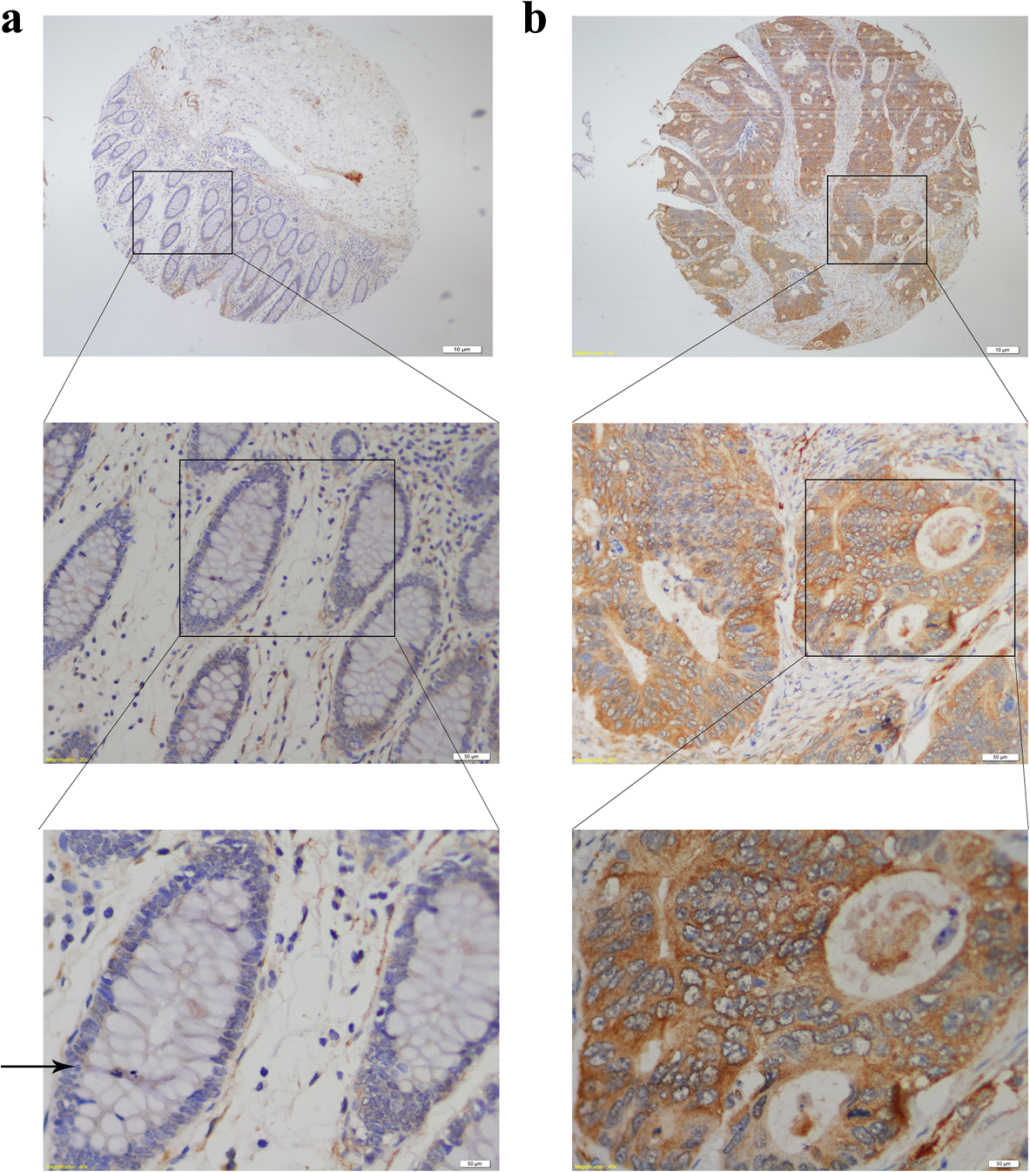
Immunohistochemical analysis of BAG3 protein expression in colorectal cancer tissues and adjacent non-tumor tissues (40×, 100× and 400×, respectively). **a** Low expression of BAG3 in adjacent non-tumor tissues. **b** High expression of BAG3 in colorectal tumor tissues

This study investigated the expression of BAG3 protein in colorectal cancer tissue specimens (*n* = 90) and matched adjacent normal colorectal tissues using immunohistochemical analysis. The analysis demonstrated that the BAG3 protein was predominantly localized in the cytoplasm of the colorectal cancer cells. BAG3 expression was significantly higher in colorectal tumor tissue than in normal colorectal tissues as shown in Fig. 1 and Table 1 (*P* = 0.000).

**Table 1:** BAG3 protein expression in colorectal cancer tissues and adjacent non-tumor tissues

Tissue type	number	BAG3 expression		χ^2^	*p*
		0–5 scores low	6–12 scores high		
Colon cancer	90	56	34	38.625	0.000
Adjacent non-tumor tissues	90	89	1		

### BAG3 protein expression and clinicopathological features of colorectal cancer patients

**Fig. 2:**
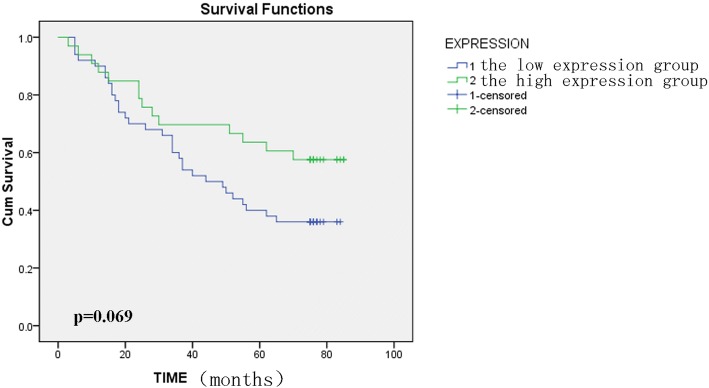
Kaplan-Meier analysis of overall survival(months) in 90 patients with high and low BAG3 expression. BAG3 protein expression in tumor tissue is not associated with colorectal cancer patient prognosis (*P* = 0.069 > 0.05)

We examined the relationship between BAG3 protein expression and clinicopathological *features* in 90 patients with colorectal cancer. BAG3 protein expression was associated with tumor size and gender (*P* = 0.001, *P* = 0.038); a greater proportion of females had tumors with high BAG3 scores than males and also patients with tumor size more than 5 cm had high BAG3 scores. While BAG3 protein expression was not associated with the patients’ age, tumor-node-metastasis stage and lymph node metastasis in this study (Table 2). Although there was a difference in patient survival between the low and high BAG3 expression groups with a tendency towards poor survival when BAG3 levels are high, this was not statistically significant, the overall median survival time is 56 months (*P* = 0.069, Fig. 2). Furthermore, univariate and multivariate survival analyses were conducted and the results indicate that age, TNM grade and lymph node metastasis were associated with the prognosis of colon cancer, age and TNM grade were isolated factors harmful to the prognosis of patients with colon cancer (Table 3).

**Table 2:** BAG3 protein expression and clinicopathological features of colorectal cancer patients

Clinicopathological feature	cases	BAG3 expression		χ2	*P* value
		0–5 scores Low, n (%)	6–12 scores High, n (%)		
Gender				4.284	0.038
male	47	34 (37.7)	13 (14.4)		
female	43	22 (24.4)	21 (23.3)		
Age				0.379	0.538
≤ 65	35	20 (22.3)	15 (16.6)		
> 65	55	35 (38.8)	20 (22.3)		
Tumor size (cm)				11.328	0.001
≤ 5	47	37 (42.0)	10 (11.4)		
> 5	41	18 (20.4)	23 (26.2)		
Tumor differentiation				4.600	0.100
I	5	5 (5.6)	0 (0)		
II	49	32 (35.6)	17 (18.9)		
III	36	19 (21.1)	17 (18.9)		
IV	0	0 (0)	0 (0)		
TNM stage				2.531	0.470
I	8	5 (5.6	3 (3.4)		
II	47	33 (37.1)	14 (15.7)		
III	32	17 (19.1)	15 (16.9)		
IV	2	1 (1.1)	1 (1.1)		
Lymph node metastasis				0.175	0.096
Negative	55	38 (42.7)	17 (19.1)		
Positive	34	18 (20.2)	16 (18.0)		

Note: There are 2 cases with no available tumor size, 1case with no available TNM stage and lymph mode metastasis, these cases are missing in the origin clinical follow-up data table which is provided by the Shanghai Outdo Biotech Company

**Table 3:** Univariate and multivariate Cox regression proportional hazards analysis

	Univariate regression			Multivariate regression		
	HR	95% CI	P –value	HR	95% CI	P –value
Sex	1.319	0.736–2.364	0.352			
Age	0.469	0.242–0.910	0.025*	2.312	1.123-4.761	0.023*
Tumor size	0.689	0.386–1.231	0.209			
Pathology classificatio	0.613	0.343–1.096	0.099			
TNM grade	0.380	0.211–0.682	0.001*	6.401	1.994-20.552	0.002*
Lymphnode metastasis	0.379	0.204–0.704	0.002*	0.315	0.076-1.307	0.112
BAG3 expression	1.774	0.945–3.33	0.075			

*P < 0.05. CI, confidence interval; HR, hazard ratio

### BAG3 overexpression promotes colorectal cancer cell growth in vitro

**Fig. 3:**
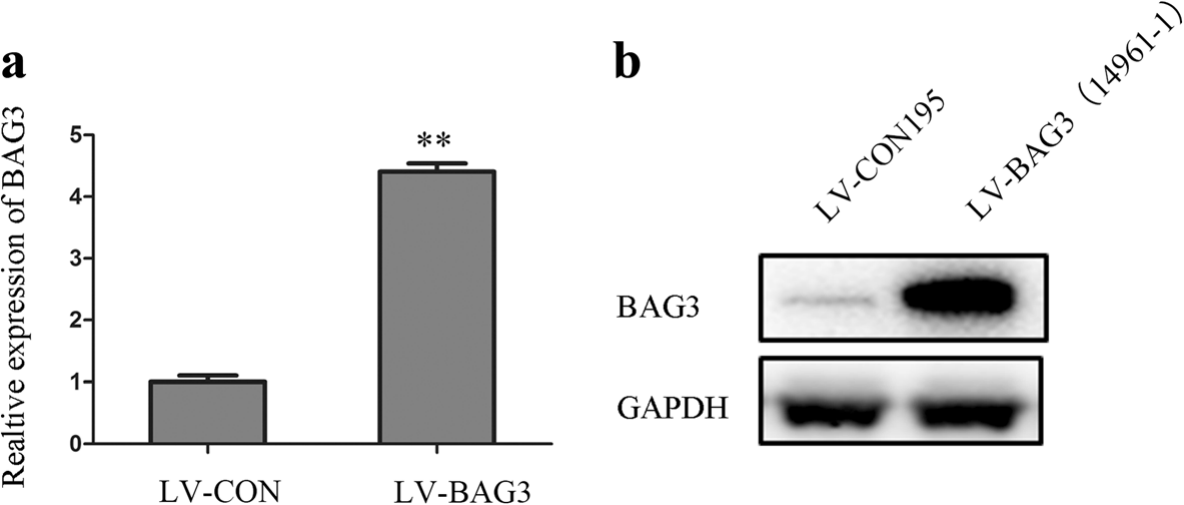
BAG3 stable overexpression in HCT-116 cells. **a** The relative expression of BAG3 mRNA in cells. **b** Western blot analysis of BAG3 overexpression in HCT-116 cells. Data represent the mean ± S.D. from three independent experiments

**Fig. 4:**
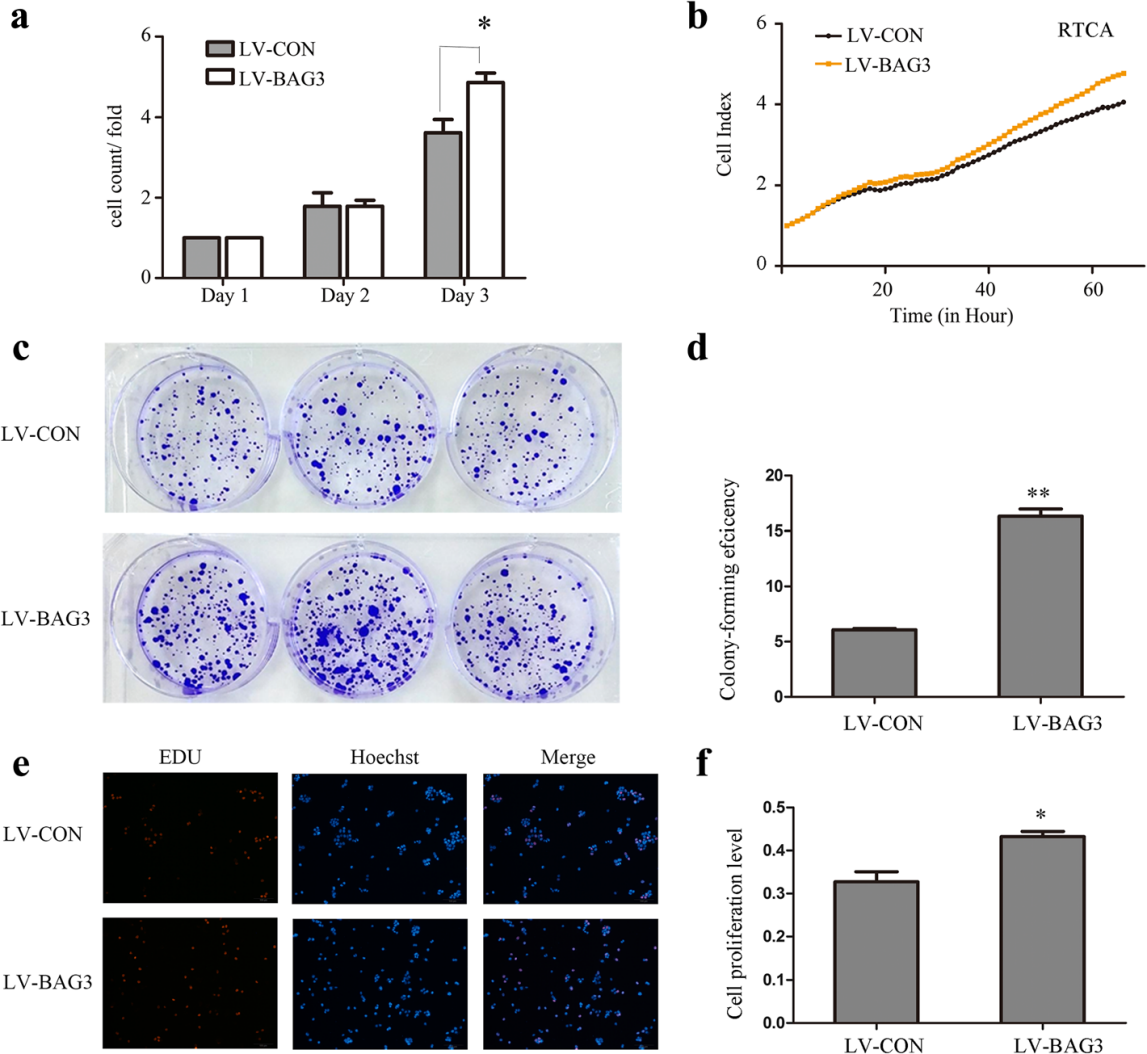
Overexpression of BAG3 promotes HCT-116 cell growth in vitro. **a** Cell counting was used to analyze cell proliferation. **b** RTCA assay was performed to record the cell survival curves. **c** Colony formation assay was performed to investigate colony formation ability in HCT-116 cells. **d** Quantitative results of colony formation analyzed with Image J. **e** EdU assay were used to examine cell viability. **f** Quantitative results of EdU assay analyzed with Image J. Data represent the mean ± S.D. from three independent experiments. *P < 0.05; **P < 0.01

We established a model of BAG3 stable over-expression in HCT-116 cells by lentiviral infection to investigate the influence of BAG3 overexpression on HCT-116 cells. After 72 h, we examined the infection efficiency using qRT-PCR and Western blot analyses. These analyses determined that BAG3 expression was markedly upregulated in BAG3 transfected HCT-116 cells compared with control cells (Fig. 3). We counted cells and performed the RTCA assay, which found that cells with BAG3 overexpression grew faster than control cells (Fig. 4a, b, *P = 0.002*). HCT-116 cells, which stably overexpressed BAG3, formed more colonies compared with control cells (Fig. 4c, d, *P = 0.000*). The Edu assay was then performed to examine the viability of BAG3 transfected HCT-116 cells. The growth of HCT-116 cells with BAG3 overexpression was significantly increased compared to control cells (Fig. 4e, f, *P = 0.000*).

### BAG3 knockout inhibits colorectal cancer cell growth in vitro

**Fig. 5:**
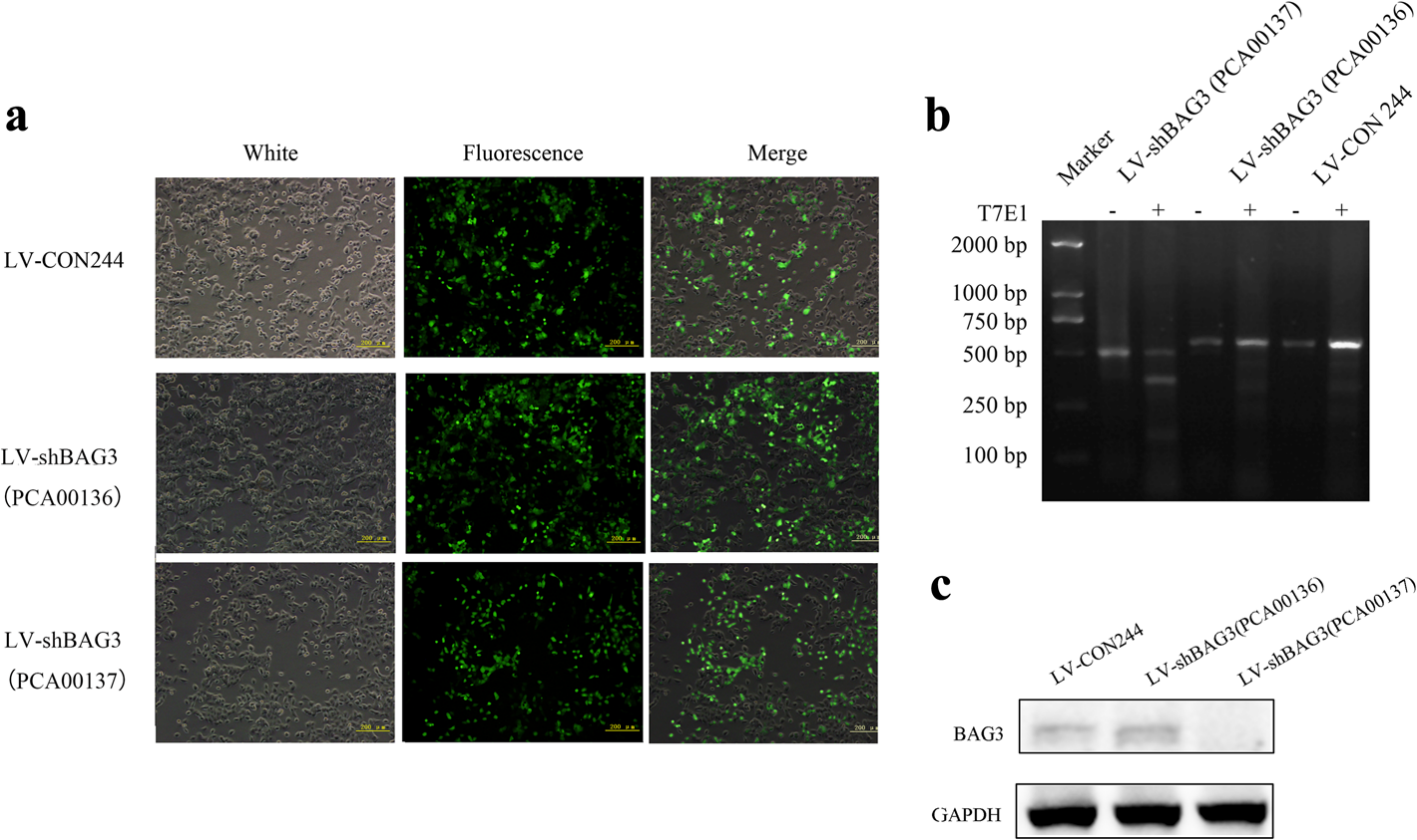
Knockout of BAG3 expression in HCT-116 cells. **a** A fluorescence microscope was used to confirm the transfection efficiency in HCT-116 cells after 72 h of lentiviral transfection. **b** DNA agarose gel electrophoresis was performed to confirm the knockout of the target gene. **c** Western blot analysis was used to examine the expression of BAG3 protein in knockout cells. Data represent mean ± S.D. from three independent experiments

**Fig. 6:**
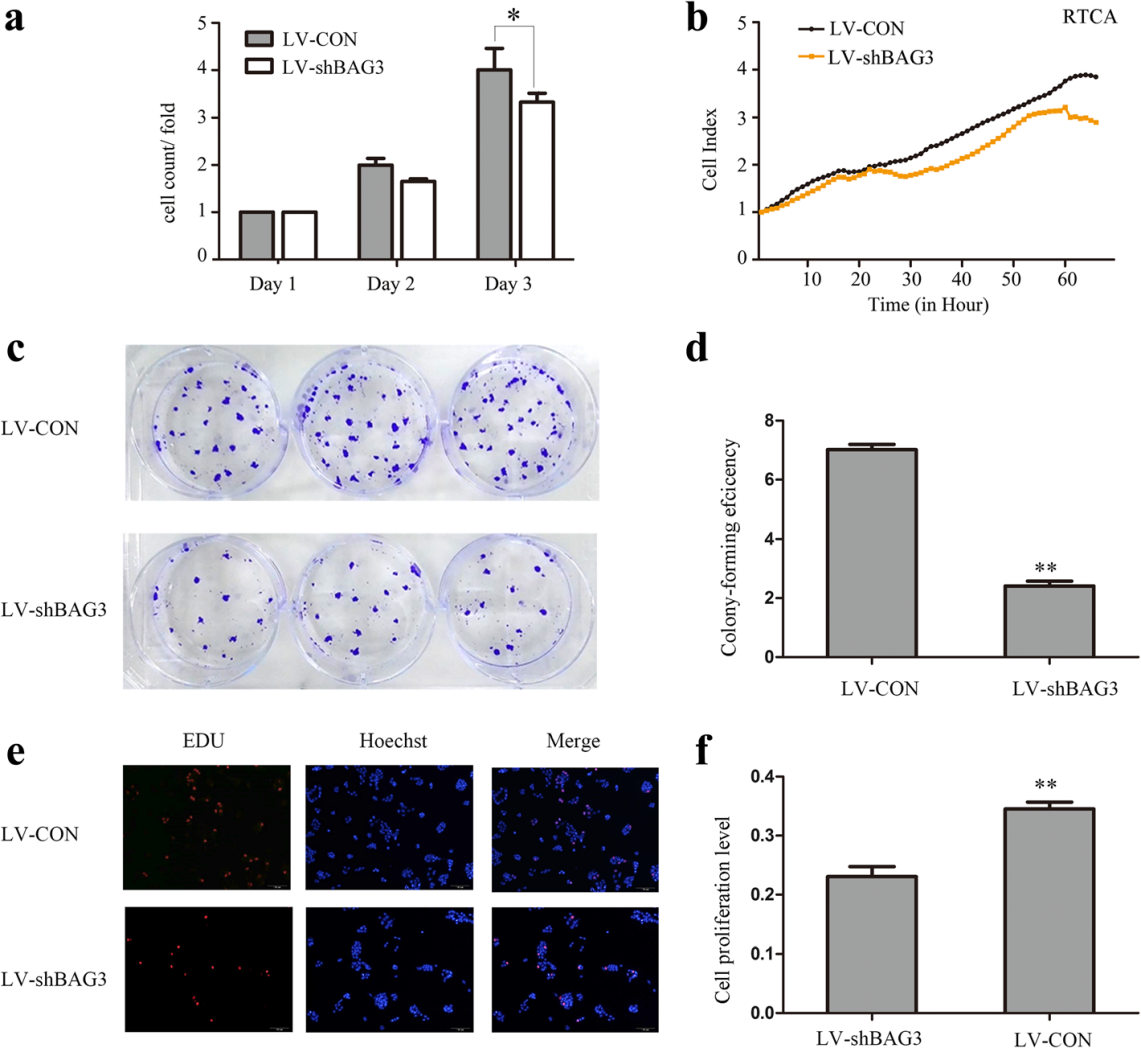
BAG3 knockout inhibits colorectal cancer cells growth in vitro*.* **a** Cell counting was used to analyze cell proliferation. **b** RTCA assay was performed to record the cell survival curves. **c** Colony formation assay was performed to investigate the colony formation ability of HCT-116 cells with BAG3 knockout. **d** Quantitative results of colony formation were analyzed with Image J. **e** EdU assay was used to examine cell viability. **f** The quantitative results of the EdU assay were analyzed with Image J. Data represent the mean ± S.D. from three independent experiments. *P < 0.05; **P < 0.01

We examined the effects of BAG3 knockout in HCT-116 cells using CRISPR/Cas9. After 72 h, infection efficiency was examined by fluorescence microscope, agrose gel electrophoresis and Western blot (Fig. 5a, b, c). As shown in Fig. 5a, the percentage of positive cells in LV-CON244, LV-shBAG3(PCA00136) and LV-shBAG3(PCA00137) groups were 67.83, 74.75 and 53.08% respectively, so the transfection efficiency was high enough for the following assays. The RTCA assay and cell counting results showed that BAG3 knockout inhibited HCT-116 cells growth (Fig. 6a, b*, P = 0.033*). Cells with lower BAG3 expression also formed fewer colonies compared with control cells (Fig. 6c, d, *P = 0.000*). Additionally, the EdU assay showed lower viability of HCT-116 cells with lower BAG3 expression (Fig. 6e, f*, P = 0.002*).

### Overexpression of BAG3 promotes colorectal cancer cells invasion and migration in vitro

**Fig. 7:**
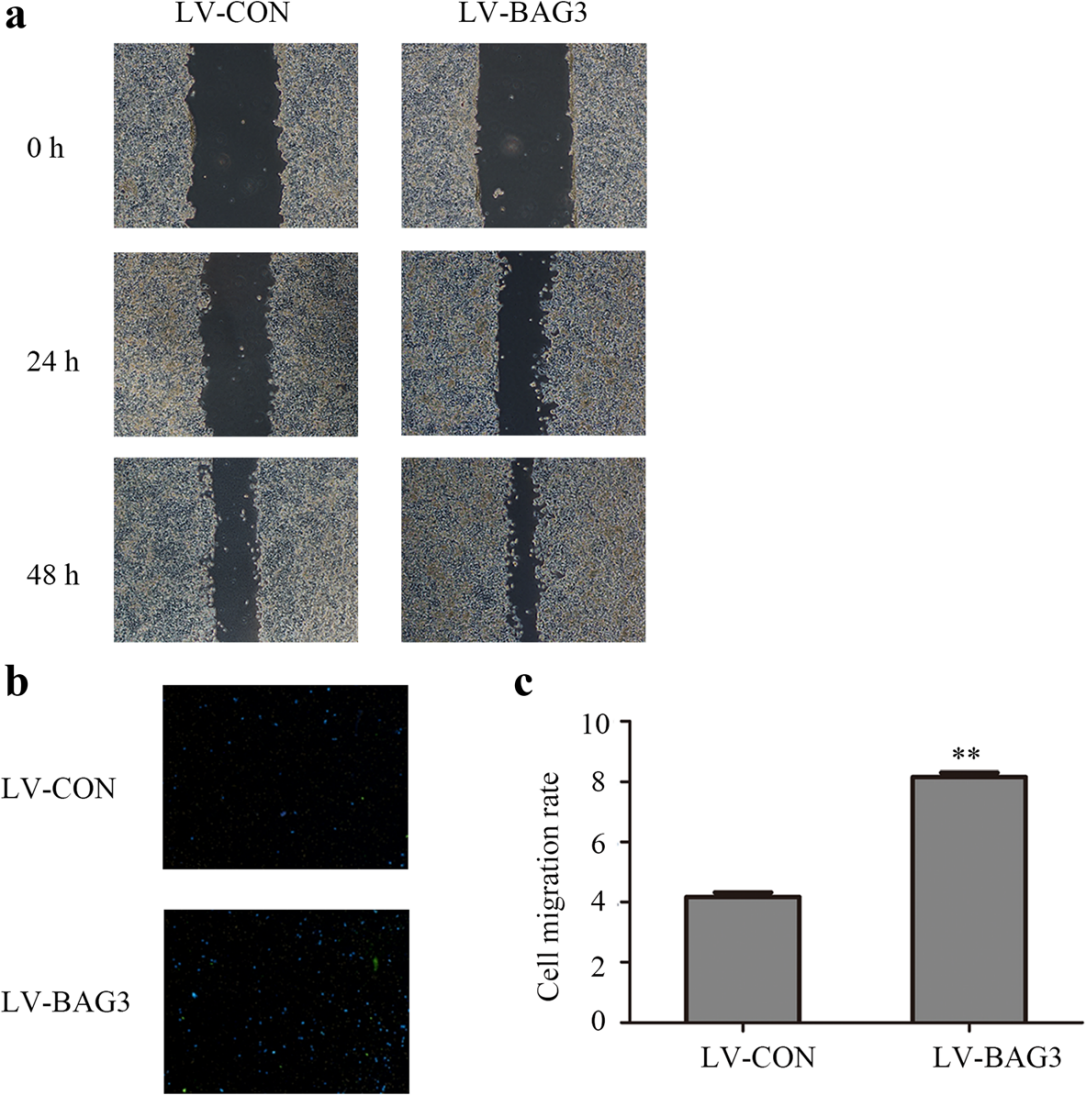
BAG3 overexpression promotes the invasion and migration of HCT-116 cells in vitro. **a** A wound-healing assay was performed to investigate the migration ability of HCT-116 cells with BAG3 overexpression. **b** A transwell invasion assay was performed to investigate the invasive ability of HCT-116 cells with BAG3 overexpression. **c** The quantitative result of the transwell invasion assay was analyzed with Image J. Data represent the mean ± S.D. from three independent experiments. *P < 0.05; **P < 0.01

**Fig. 8:**
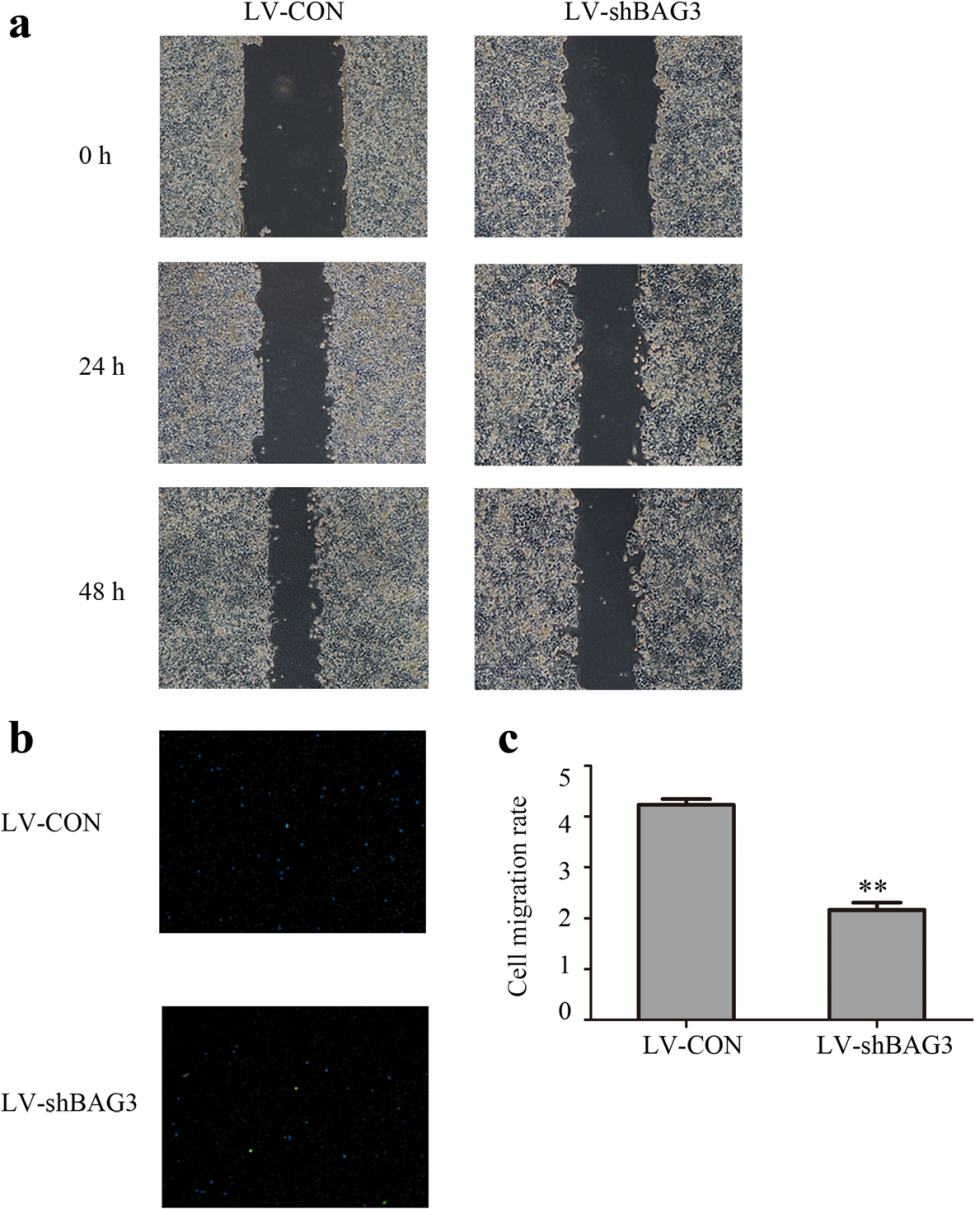
BAG3 knockout inhibits the invasion and migration of HCT-116 cells in vitro. **a** A wound-healing assay was performed to investigate the invasive ability of HCT-116 cells. **b** A transwell assay was performed to investigate the invasive ability of HCT-116 cells. **c** The quantitative result of the transwell assay was analyzed with Image J. Data represent the mean ± S.D. from three independent experiments. *P < 0.05; **P < 0.01

We measured the migration and invasion ability of HCT-116 cells with BAG3 overexpression using wound-healing and transwell invasion assays. The wound-healing assay results showed that scratch wounds in HCT-116 cells with BAG3 overexpression healed significantly faster compared with the control cells (Fig. 7a), while HCT-116 cells with BAG3 knockout showed slower wound healing compared with the control cells (Fig. 8a). Transwell invasion assay showed that increased BAG3 expression enhanced HCT-116 cells invasion ability compared with control cells (Fig. 7b and c, *P* < 0.05,). BAG3 knockout significantly inhibited the invasion ability of HCT-116 cells (Fig. 8b, c, P < 0.05). Taken together, these results indicate that BAG3 can promote colorectal cancer cell invasion and migration in vitro.

### Influence of BAG3 expression level on chemoresistance in HCT-116 cells

**Fig. 9:**
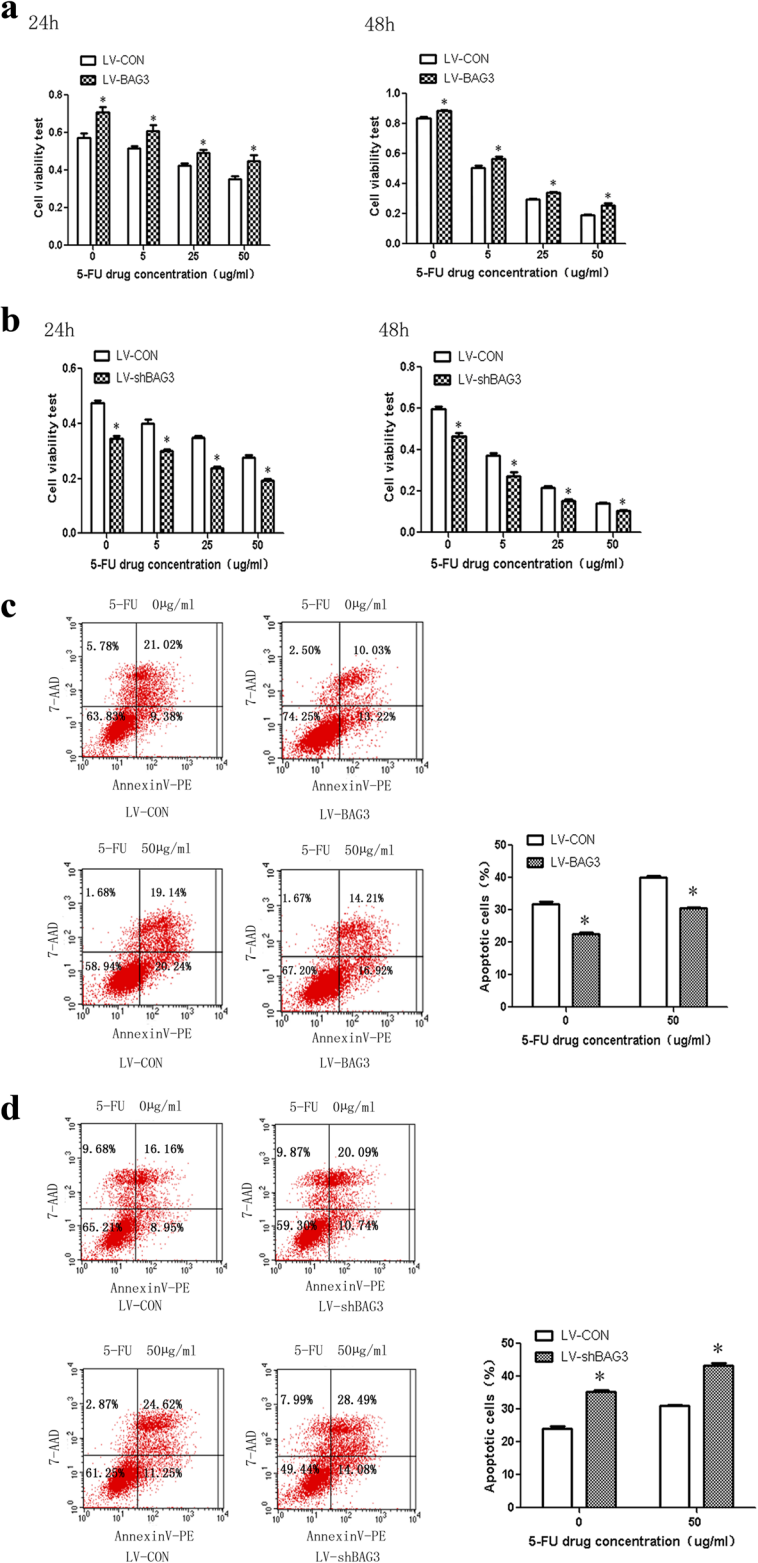
Influence of BAG3 expression levels on HCT-116 chemoresistance to 5-FU treatment. HCT-116 cells with BAG3 overexpression (**a**) or BAG3 knokout (**b**) treated with 5-FU in different concentrations and duration. MTT assay was used to detect cell viability. The apoptosis level in HCT-116 cells with BAG3 overexpression (**c**) or knockout (**d**) and control treated with 5-FU in different concentrations and duration. *P < 0.05

To the best of our knowledge, the effects of BAG3 expression on chemoresistance have not yet been reported in colorectal cancer. Therefore, we examined these effects in HCT-116 cells in vitro. As shown in Fig. 9a, the cell viability was significantly higher in HCT116 cells with BAG3 overexpression compared to control cells treated with 0,5, 25, and 50 μg/ml 5-FU for 24 h or 48 h. However, cell viability tended to decrease in HCT-116 cells with BAG3 knockout compared to control cells treated with 0, 5, 25, or 50 μg/ml 5-FU for 24 h or 48 h, as shown in Fig. 9b.

We used flow cytometry to examine the level of apoptosis in different treatment groups. HCT-116 cells with BAG3 overexpression had lower apoptosis levels compared to the control cells in this study, while the HCT-116 cells with BAG3 knockout had higher levels of apoptosis than the control cells treated with 0 and 50 μg/ml 5-FU for 48 h, as shown in Fig. 9c and d.

### Key signaling pathways in HCT-116 cells affected by BAG3 knockout

**Fig. 10:**
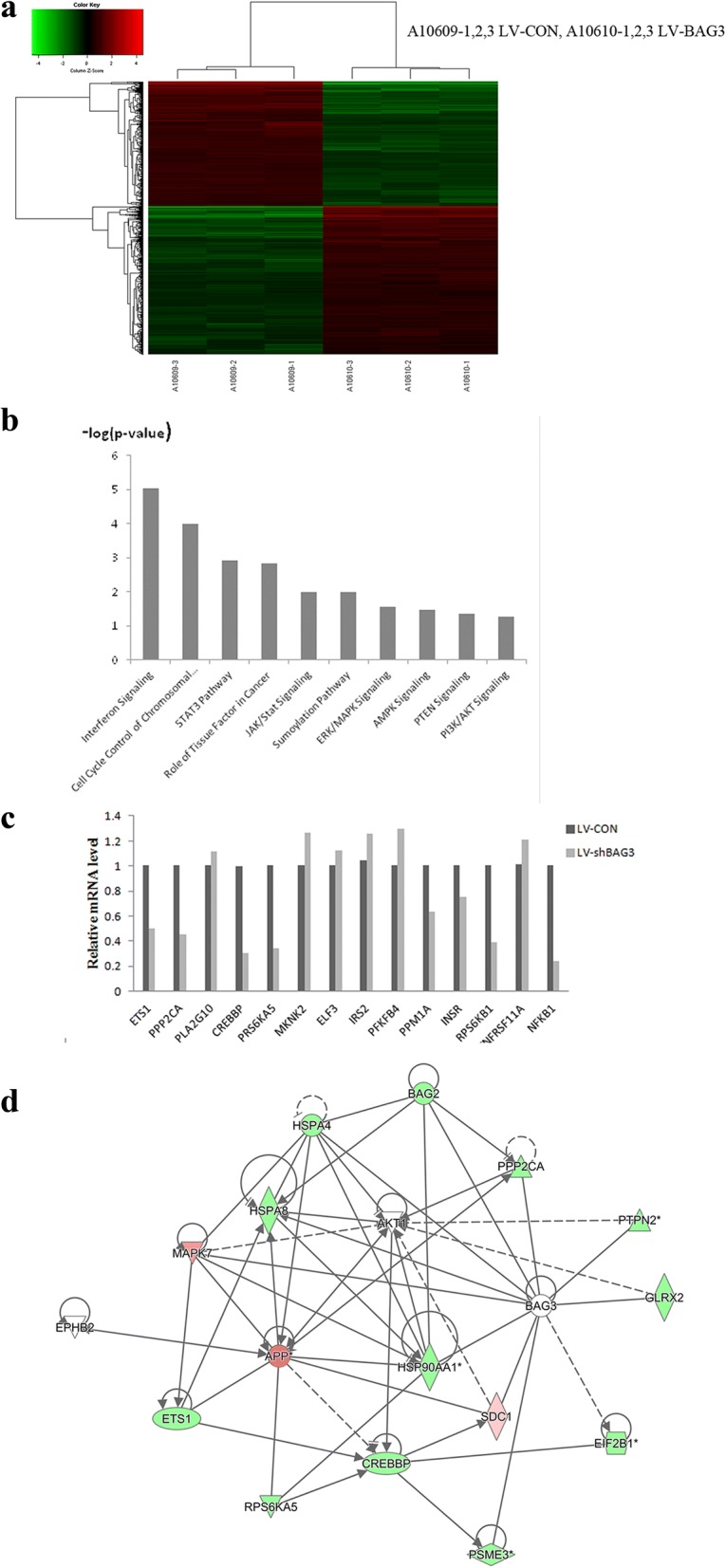
Microarray analysis of changes in the gene expression and signaling pathways affected by BAG3 knockout in HCT-116 cells. **a** Heat map representation of 1224 genes with significant differential expression in HCT-116 cells with or without BAG3 knockout. **b** Functional pathway enrichment analysis using IPA. **c** Microarray data were further confirmed by qRT-PCR analysis of selected genes. **d** Gene interaction network in HCT-116 cells after BAG3 knockout. Green represents downregulated genes, while red represents upregulated genes. The solid lines represent direct interactions, while the dotted lines represent indirect interactions

To gain further insight into the molecular mechanisms underlying the function of BAG3 in colon cancer, we performed global gene expression profiling of HCT-116 cells with or without BAG3 knockout, as shown in Fig. 10a. In our model, 653 genes were upregulated and 571 genes were downregulated by BAG3 knockout. Functional properties of differentially expressed genes were analyzed using IPA, which demonstrated that these genes were enriched in the interferon signaling pathways as well as in the JAK/Stat, ERK/MAPK, AMPK PTEN, and PI3K/AKT signaling pathways. We then confirmed via quantitative real-time PCR that BAG3 knockout induced changes in the expressions of genes related to the ERK/MAPK pathway (ETS1, PPP2CA, PLA2G10, CREBBP, RPS6KA5, MKNK2, and IRS2), AMPK pathway (PFKFB4, PPM1A, and INSR) and PTEN pathway (RPS6KB1, TNFRSF11A, and NFKB1) (Fig. 10b, c). We performed IPA network analysis and found gene interaction networks involved in the regulation of BAG3 in HCT-116 cells (Fig. 10d).

## Discussion

BAG3 protein is an anti-apoptotic protein involved in many other cellular processes, including cell cycle regulation, cell survival, proliferation, adhesion, migration, autophagy and EMT [5, 7, 10, 16, 22, 28]. Some studies have shown that BAG3 can regulate these processes via the K-ras [29], EGFR [30] or AMPK/PI3K [31] signaling pathways. Studies have found that BAG3, a member of the co-chaperone family, contains the BAG domain, which can interact with the ATPase domain of heat shock protein 70 to regulate the HSP70 pathway [32–34].

In normal cells, BAG3 expression is very low, while its expression is higher in many neoplastic cell types including leukemias and lymphomas, solid tumors such as melanoma, glioblastoma, pancreatic carcinomas and thyroid carcinomas [5, 7, 35, 36]. Previous studies demonstrated that the overexpression of BAG3 can inhibit apoptosis and promote the survival of certain types of leukemia cells [37, 38]. Past reports asserted that the BAG3 protein interacts with Bcl-2 to enhance the activity of Bcl-2, thereby promoting cell survival and metastasis [39, 40].

Despite previous reports on BAG3 expression in tumors, few studies have examined BAG3’s role in colorectal cancer [41, 42]. This study systematically examined the effects of BAG3 protein overexpression and knockout in human colon cancer HCT-116 cells for the first time and provided evidence of BAG3 as a potential therapeutic target against colorectal cancer.

We first examined BAG3 protein expression in clinical samples (tumor and matched adjacent non-tumor tissues) from colorectal cancer patients using immunohistochemistry. BAG3 protein expression was significantly higher in colorectal cancer tissues than in matched adjacent non-tumor tissues, which indicated that BAG3 protein may participate in CRC tumorigenesis and progression. We then analyzed the relationship between BAG3 expression and patient clinicopathological parameters and found that BAG3 expression was closely related to the tumor size and patients’ gender (*P* < 0.05), though differences in BAG3 expression did not affect patient prognosis. A recent study [43] found that BAG3 can bind to a specific receptor, such as IFITM2, expressed on macrophages, and induce the release of factors that sustain tumor growth and metastasis. In 2013, Brendel A et al. [44] also investigated potential targets of the ER subtype alpha and found that prosurvival BAG3 expression was highly upregulated in the presence of ERalpha. These findings may explain why BAG3 expression in our study was different with respect to tumor size and gender. Furthermore, Cox Univariate and multivariate regression analysis indicated that age, TNM stage and Lymphnode metastasis were related to survival time, and high BAG3 expression had a tendency toward poor survival, which was supported by a statistical trend and should be investigated further in the future with more patient samples.

We established in vitro models of BAG3 overexpression and knockout in HCT-116 cell line to further clarify the role of BAG3 in colorectal cancer tumorigenesis. By performing cell counting, colony formation assays, RTCA assays, EdU cell proliferation assays, wound-healing and transwell matrigel assays, we demonstrated that BAG3 overexpression promoted HCT-116 cell proliferation, colony formation, migration and invasion, while BAG3 knockout inhibited these processes. These findings suggest that BAG3 plays a role in the progression and metastasis of colorectal cancer.

The association between BAG3 expression and chemoresistance has not yet been examined in colorectal cancer. Therefore, this is the first study investigating the role of BAG3 in colon cancer chemoresistance. HCT-116 cells with high expressions of BAG3 had higher cell viability and lower apoptosis rate compared with control cells in this study. HCT-116 cells with BAG3 knockout had lower cell viability and higher apoptosis rate compared with control cells after treatment with different concentrations of 5-FU for 24 h and 48 h. Collectively, these results indicate that BAG3 is involved in 5-FU resistance in HCT-116 cells. Another study [45] showed that BAG3 silencing promotes the sensitivity of ovarian cancer cells to cisplatin. A growing number of studies recently confirmed that the activation of autophagy contributes to chemoresistance in cancer cells and that the downregulation of autophagy sensitizes cancer cells to therapeutic drugs. Consistent with other research, we confirmed that silencing BAG3 significantly increases 5-FU induced apoptosis. The underlying molecular mechanisms regulating the sensitivity of colon cancer cells to 5-FU by BAG3, a novel regulator of autophagy, should be further investigated.

In accordance with the functional results on the role of BAG3 in colorectal cells in vitro described above, our microarray analysis revealed that corresponding pathways were also dysregulated in HCT-116 cells with BAG3 knockout. 653 genes were upregulated and 571 genes were downregulated by BAG3 knockout in this study. The functional characteristics of these differentially expressed genes were analyzed using Ingenuity Pathway Analysis, which demonstrated that the genes were enriched in pathways such as JAK/Stat, ERK/MAPK, AMPK, PTEN and PI3K/AKT.

In addition to pathway enrichment analyzed for BAG3-associated gene sets, the expressions of several cell proliferation and survival-related genes were confirmed by qRT-PCR. These genes belong to three signaling pathways: the ERK/MAPK pathway (ETS1, PPP2CA, PLA2G10, CREBBP, RPS6KA5, MKNK2, ELF3, and IRS2), AMPK pathway (PFKFB4, PPM1A, and INSR) and PTEN pathway (RPS6KB1, TNFRSF11A, and NFKB1). PLA2G10, MKNK2, ELF3, IRS2, PFKFB4, and TNFRSF11A were upregulated, while other genes were downregulated. Based on IPA network analysis, we speculate that there is a core axis, the AKT-MAPK axis, and that PPP2CA can influence the MAPK signaling pathway through AKT. RPS6KA5, CREBP and ETS1 may be the target gene influenced by BAG3 knockout, which could impact HCT-116 cell proliferation, migration and chemoresistance.

## Conclusions

This study demonstrates that the expression of BAG3 in colorectal cancer tissue is higher than in non-tumor tissue in the same patient. BAG3 overexpression in colorectal cancer can promote tumor proliferation, migration and invasion, while BAG3 knockout can inhibit these processes. BAG3 can also cause 5-FU-resistance in HCT-116 cells, while knockout of BAG3 sensitized HCT-116 cells to 5-FU. Microarrays and network analyses further clarified the signaling pathways involved in these cellular effects of BAG3. Therefore, we conclude that BAG3 may be used as a potential biomarker or therapeutic target for colorectal cancer.

## Abbreviations

5-FU: 5-fluorouracil
7-AAD: 7-amino-actinomycin D
AMPK: Adenosine 5′-monophosphate (AMP)-activated protein kinase
BAG3: Bcl-2-associated athanogene 3
Bcl-2: B-cell lymphoma-2
CRC: colorectal cancer
CREBBP: CREB binding protein
DMSO: Dimethyl Sulfoxide
EdU: 5-Ethynyl-2′-deoxyuridine
EGFR: epidermal growth factor receptor
EMT: Epithelial-Mesenchymal Transition
ER: Endoplasmic reticulum;
ERK: extracellular regulated protein kinases
ETS1: ETS proto-oncogene 1
FACS: Fluorescence activated cell sorting
GAPDH: glyceraldehyde-3-phosphate dehydrogenase
HSP70: Heat shock protein 70
IFITM2: Interferon-induced transmembrane protein 2
INSR: insulin receptor
IRS2: insulin receptor substrate 2
JAK: Janus kinas
MAPK: mitogen-activated protein kinase
MKNK2: MAP kinase interacting serine/threonine kinase 2
NFKB1: nuclear factor kappa B subunit 1
PFKFB4: 6-phosphofructo-2-kinase/fructose-2,6-biphosphatase 4
PI3K: phosphatidylinositol 3-kinase
PLA2G10: phospholipase A2 group X
PPM1A: protein phosphatase, Mg2+/Mn2+ dependent 1A
PPP2CA: protein phosphatase 2 catalytic subunit alpha
PTEN: gene of phosphate and tension homology deleted on chromsome ten
qRT-PCR: quantitative real-time PCR
RPS6KA5: ribosomal protein S6 kinase A5
RPS6KB1: ribosomal protein S6 kinase B1
RTCA: Real-time cell analyzer
SDS-PAGE: sodium dodecyl sulfate polyacrylamide gel electrophoresis
STAT: Signal transducers and activators of transcription
TNFRSF11A: TNF receptor superfamily member 11a
